# Prevalence of Diabetes Medication Intensifications in Older Adults Discharged From US Veterans Health Administration Hospitals

**DOI:** 10.1001/jamanetworkopen.2020.1511

**Published:** 2020-03-24

**Authors:** Timothy S. Anderson, Sei Lee, Bocheng Jing, Kathy Fung, Sarah Ngo, Molly Silvestrini, Michael A. Steinman

**Affiliations:** 1 Division of General Medicine, Beth Israel Deaconess Medical Center, Boston, Massachusetts; 2 San Francisco VA Medical Center, San Francisco, California; 3 Division of Geriatrics, University of California, San Francisco

## Abstract

**Importance:**

Elevated blood glucose levels are common in hospitalized older adults and may lead clinicians to intensify outpatient diabetes medications at discharge, risking potential overtreatment when patients return home.

**Objective:**

To assess how often hospitalized older adults are discharged with intensified diabetes medications and the likelihood of benefit associated with these intensifications.

**Design, Setting, and Participants:**

This retrospective cohort study examined patients aged 65 years and older with diabetes not previously requiring insulin. The study included patients who were hospitalized in a Veterans Health Administration hospital for common medical conditions between 2012 and 2013.

**Main Outcomes and Measures:**

Intensification of outpatient diabetes medications, defined as receiving a new or higher-dose medication at discharge than was being taken prior to hospitalization. Mixed-effect logistic regression models were used to control for patient and hospitalization characteristics.

**Results:**

Of 16 178 patients (mean [SD] age, 73 [8] years; 15 895 [98%] men), 8535 (53%) had a preadmission hemoglobin A_1c_ (HbA_1c_) level less than 7.0%, and 1044 (6%) had an HbA_1c_ level greater than 9.0%. Overall, 1626 patients (10%) were discharged with intensified diabetes medications including 781 (5%) with new insulins and 557 (3%) with intensified sulfonylureas. Nearly half of patients receiving intensifications (49% [791 of 1626]) were classified as being unlikely to benefit owing to limited life expectancy or already being at goal HbA_1c_, while 20% (329 of 1626) were classified as having potential to benefit. Both preadmission HbA_1c_ level and inpatient blood glucose recordings were associated with discharge with intensified diabetes medications. Among patients with a preadmission HbA_1c_ level less than 7.0%, the predicted probability of receiving an intensification was 4% (95% CI, 3%-4%) for patients without elevated inpatient blood glucose levels and 21% (95% CI, 15%-26%) for patients with severely elevated inpatient blood glucose levels.

**Conclusions and Relevance:**

In this study, 1 in 10 older adults with diabetes hospitalized for common medical conditions was discharged with intensified diabetes medications. Nearly half of these individuals were unlikely to benefit owing to limited life expectancy or already being at their HbA_1c_ goal.

## Key Points

**Question:**

How often are older adults discharged with changes to their outpatient diabetes medication regimens following hospitalization for common medical conditions?

**Findings:**

In this cohort study of 16 178 older adults hospitalized in the Veterans Health Administration national health system, 1 in 10 patients was discharged with intensified diabetes medications. Nearly half of patients receiving intensifications had already reached outpatient blood glucose goals or had limited life expectancy.

**Meaning:**

During hospitalization, consideration of long-term diabetes control is needed in addition to inpatient blood glucose recordings to reduce potentially nonbeneficial medication changes when older adults are discharged home.

## Teaser

This cohort study estimates the prevalence of diabetes medication intensification and the likelihood of benefit associated with medication intensification among older adults hospitalized in a Veterans Health Administration hospital.

## Introduction

During hospitalization, outpatient medication regimens are frequently modified by inpatient clinicians.^1,2,3^ While medication changes are often related to the condition that led to hospitalization (eg, receiving antiplatelets following an acute myocardial infarction), inpatient monitoring may lead to adjustments of medication regimens prescribed for chronic diseases, such as diabetes and hypertension, that are not directly connected to the primary condition for which the patient was hospitalized. Nonessential modification of chronic disease regimens during hospitalization may risk medication confusion and adverse drug events if patients are discharged with prescriptions for those modified regimens.

Prior research indicates that intensifications of hypertension regimens are common in hospitalized older adults and are driven by inpatient measurements.^3,4^ Similar to blood pressure, blood glucose levels are monitored frequently in hospitalized patients with diabetes and elevated inpatient recordings may lead clinicians to discharge patients with prescriptions for intensified diabetes medications. Despite controversy over the benefit of strict inpatient glycemic control,^5,6,7^ the frequency of changes to outpatient diabetes regimens following hospitalization is unknown. For patients with severely uncontrolled diabetes (eg, hemoglobin A_1c_ [HbA_1c_] level >9.0% [to convert to proportion of total hemoglobin, multiply by 0.01]), medication intensification at discharge may improve hyperglycemia symptoms and set them on the path toward improved long-term glycemic control. In contrast, intensifications of diabetes medication regimens for patients with previously well-controlled diabetes may contribute to unnecessary polypharmacy and pose a risk of overtreatment. Both overtreatment and medication confusion may risk hypoglycemia,^8^ particularly when insulins and sulfonylureas are intensified.^9^

Understanding the impact of hospitalization on outpatient diabetes control is particularly crucial for older adults, more than 25% of whom have diabetes. Older adults are the most frequently hospitalized age group, and the balance of risks and benefits from strict blood glucose control may vary owing to limited life expectancy and elevated risks of hypoglycemia, polypharmacy, and adverse drug events.^10^ Thus, we examined a national cohort of hospitalized older adults with diabetes not previously requiring insulin to assess how often outpatient diabetes medications were intensified at hospital discharge, to identify which medications were most commonly intensified, and to determine whether life expectancy or prior outpatient diabetes control were associated with intensification decisions.

## Methods

We conducted a retrospective cohort study of older adults admitted to Veterans Affairs Administration (VHA) hospitals using VHA clinical and pharmacy data merged with VHA and Medicare claims data. This research was approved by the institutional review boards of the San Francisco Veterans Affairs Medical Center and University of California, San Francisco. A waiver of informed consent was obtained because administrative data were used and all data were deidentified. Data analysis was performed from October 20, 2018, to September 1, 2019. This study followed the Strengthening the Reporting of Observational Studies in Epidemiology (STROBE) reporting guideline.

### Study Population

The study cohort consisted of all patients aged 65 years and older with a diagnosis of diabetes who were not taking insulin prior to admission, received regular VHA outpatient care, and were admitted to a VHA hospital between January 1, 2011, and December 31, 2013, for common medical conditions.

We defined diabetes as at least 2 outpatient diagnoses or 1 hospital discharge diagnosis of diabetes in the 2 years preceding the index hospitalization.^11,12^ Algorithms based on administrative codes may capture patients with a history of diabetes, so we examined only patients who were taking a diabetes medication prior to hospitalization and those who were not taking a diabetes medication but who had an HbA_1c_ level greater than 6.5% in the year preceding admission.

Discharge diagnoses were identified by primary diagnosis code grouped by Clinical Classification Software categories^13^ and included medical and cardiovascular conditions. Medical conditions included asthma, chronic obstructive pulmonary disease, pneumonia, sepsis, skin infection, and urinary tract infection. Cardiovascular conditions included arrhythmia, chest pain, coronary artery disease, acute coronary syndrome, conduction disorders, congestive heart failure, heart valve disorder, stroke, and transient ischemic attack. These conditions were chosen as they are among the most common reasons for hospitalization and their acute management does not typically require intensification of outpatient diabetes medications. We excluded patients with secondary discharge diagnoses of diabetic ketoacidosis or hyperglycemic hyperosmolar syndrome, which might necessitate an acute change in diabetes treatment.

To ensure accurate capture of medication use, we limited our sample to patients who received at least 80% of their outpatient care in VHA settings and thus were expected to regularly receive medications from VHA pharmacy sources, and we excluded patients admitted from or discharged to an institutional setting and those admitted to the hospital in the preceding 30 days.^14^ As insulin is given by injection and instructions to change insulin doses are often not accompanied by a new prescription, dosing changes cannot be accurately assessed using pharmacy databases, so we limited our study to patients not taking insulin prior to hospitalization. Detailed exclusion criteria are provided in the eFigure in the Supplement.

### Identifying Medication Intensifications

Our primary outcome was whether a patient received 1 or more intensified outpatient diabetes medications at hospital discharge. We examined all major diabetes medication classes in use during our study period, which included sulfonylureas, biguanides, thiazolidinediones, α-glucosidase inhibitors, dipeptidyl peptidase-4 inhibitors, meglitinides, glucagon-like peptide agonists, and insulins.

We identified intensifications based on a previously validated algorithm comparing medications in use prior to hospital admission with medications prescribed at hospital discharge.^3,15^ We defined medications in use at hospital admission as the latest pharmacy fill being for a quantity sufficient to last until at least 60 days before admission. Discharge medications were defined as prescriptions filled by the outpatient VHA pharmacy between 2 days before and 2 days after discharge and prescriptions filled by the inpatient pharmacy on the day of discharge with a supply of at least 7 days.

Medication intensifications were defined to include both new medication additions and dose increases of admission medications. Medications not present on admission but prescribed at discharge were classified as additions. Admission medications for which a discharge prescription was filled for greater than 20% of the preadmission dose were classified as dose increases.

### Covariates

The primary variables evaluated for association were preadmission and inpatient diabetes control. Preadmission diabetes control was measured using the most recent HbA_1c_ laboratory value measured within 1 year prior to hospitalization and categorized as tightly controlled (HbA_1c_ level <7.0%), controlled (HbA_1c_ level 7.0%-8.9%), elevated (HbA_1c_ level >9.0%), or not measured in prior year. Inpatient diabetes control was measured by inpatient blood glucose recordings and categorized as severely elevated (>3 recordings >299 mg/dL [to convert to millimoles per liter, multiply by 0.0555]), moderately elevated (>3 recordings >199 mg/dL), or not elevated*.*

Covariates included age, sex, ethnicity, median household income estimated from residential zip codes, counts of diabetes and overall admission medications, preadmission adherence to diabetes medications defined by composite proportion of days covered in the prior year, individual conditions of the Charlson Comorbidity Index (calculated from VHA and Medicare claims during the 24 months before hospitalization),^16^ and outpatient kidney function measured by estimated glomerular filtration rate.^17^ Hospitalization-related covariates included year of admission, training hospital status, length of stay, primary discharge diagnosis, inpatient glomerular filtration rate measured on the hospital day closest to discharge, and an indicator variable for receipt of inpatient corticosteroid medications, as corticosteroids are associated with elevated blood glucose levels.^18^

### Statistical Analysis

We present unadjusted proportions of patients who received diabetes medication intensifications overall, by medication class, and by intensification type. We describe the proportion of patients receiving high-risk intensifications, defined as receipt of intensified insulin or sulfonylurea medications, as these medications are more strongly associated with severe hypoglycemia than other diabetes medications.

Based on prior research and national guidelines that recommend less strict HbA_1c_ targets in older adults with limited life expectancy,^10,19,20,21^ we examined the proportion of patients discharged with intensifications who were likely or unlikely to benefit from stricter glycemic control based on preadmission HbA_1c_ and estimated life expectancy. Estimated life expectancy was calculated from age and Charlson Comorbidity Index score and categorized as less than 5 years, 5 to 10 years, and greater than 10 years, as in prior studies.^22,23^ Patients were categorized as likely to benefit if their preadmission HbA_1c_ level was greater than 9.0%. Patients were categorized as unlikely to benefit if their preadmission HbA_1c_ level was less than 7.5% regardless of life expectancy or if their preadmission HbA_1c_ level was less than 9.0% and estimated life expectancy was less than 5 years. All others were classified as indeterminate.

We conducted multivariable mixed-effect logistic regression analyses to determine associations between diabetes medication intensification and preadmission and inpatient diabetes control. Adjusted analyses included the covariates we have noted, a random-effect term to account for clustering by hospital, and an interaction term to account for the association between preadmission and inpatient diabetes control. Missing data were imputed using iterative Markov chain Monte Carlo method and 10 imputation sets. We used postestimation margins following regression analyses to calculate predicted probabilities of intensification by preadmission and inpatient diabetes control and likelihood to benefit category.

We determined statistical significance by using 95% confidence intervals and 2-tailed tests with *P* < .05 as the threshold for significance. We used Stata version 14.1 (StataCorp) for all analyses.

## Results

We identified 16 178 older adults (mean [SD] age, 73 [8] years; 15 895 [98%] men) with diabetes who were discharged from VHA medical centers (Table 1). Nearly 70% of patients were taking 1 or more diabetes medications prior to hospitalization, with metformin and sulfonylureas most commonly used. More than one-quarter of patients had an estimated life expectancy less than 5 years. The most common discharge diagnoses were congestive heart failure (16%), pneumonia (12%), coronary artery disease (11%), conduction disorders (10%), and chronic obstructive pulmonary disease (10%).

**Table 1.** Cohort Characteristics^a^

Characteristic	Patients, No. (%) (N = 16 178)
**Demographic and comorbidity**	
Age, median (IQR), y	73 (67-80)
Female	283 (2)
Race/ethnicity	
White	13 138 (81)
Black	2336 (14)
Hispanic	225 (1)
Other	479 (3)
Income, mean (SD), $	26 653 (46 601)
Selected comorbidities	
Congestive heart failure	6722 (42)
Kidney disease	5061 (31)
Cerebrovascular accident	4217 (26)
Prior myocardial infarction	3024 (20)
Malignant neoplasm	3574 (22)
Dementia	519 (3)
Estimated life expectancy, y^b^	
>10	7065 (44)
5-10	4652 (29)
<5	4461 (28)
**Hospitalization **	
Year of hospitalization	
2011	6370 (39)
2012	5101 (32)
2013	4707 (29)
Training hospital	14 551 (90)
Length of stay, median (IQR), d	4 (2-6)
Discharge diagnosis	
Arrhythmia	217 (1)
Asthma	84 (1)
Chronic obstructive pulmonary disease	1531 (10)
Chest pain	664 (4)
Conduction disorders	1617 (10)
Coronary artery disease	1795 (11)
Acute coronary syndrome	971 (6)
Congestive heart failure	2553 (16)
Heart valve disorder	312 (2)
Pneumonia	1893 (12)
Sepsis	285 (2)
Skin infection	1377 (9)
Stroke	791 (5)
Transient ischemic attack	263 (2)
Urinary tract infection	1429 (9)
Venous thromboembolism	396 (3)
Estimated glomerular filtration rate, mean (SD), mL/min/1.73 m^2^	
Preadmission	66 (24)
Discharge	68 (27)
**Medication use**	
No. of admission medications, median (IQR)	9 (6-12)
No. of admission diabetes medications	
0	4153 (26)
1	8263 (51)
2	3350 (21)
≥3	412 (3)
Admission diabetes medications	
Sulfonylureas	7830 (48)
Biguanides	7328 (45)
Thiazolidinediones	535 (3)
α-Glucosidase inhibitors	265 (2)
Dipeptidyl peptidase-4 inhibitors	145 (1)
Meglitinides	28 (0)
Glucagon-like peptide agonists	5 (0)
Prior diabetes medication adherence, proportion of days covered, %^c^	
<80	3700 (23)
≥80	8325 (52)
Not taking diabetes medications at admission	4153 (26)
Any receipt of inpatient corticosteroids	1810 (11)

Abbreviation: IQR, interquartile range.

^a^ Multiple imputation was used to account for missing data, which included inpatient estimated glomerular filtration rate (1300 patients), outpatient estimated glomerular filtration rate (1356 patients), and inpatient glucose recordings (673 patients).

^b^ Life expectancy was calculated based on number of comorbidities and age.

^c^ Adherence was calculated from electronic pharmacy dispensing data as the proportion of days covered for each admission diabetes medication in the year prior to index hospital admission. A threshold of 80% is a commonly used criterion to determine clinically significant nonadherence. To account for patients taking multiple diabetes mediations prior to hospitalization, a composite proportion of days covered was calculated as the mean of each individual diabetes medication proportion of days covered.

### Association Between Preadmission and Inpatient Diabetes Control

**Figure 1.**
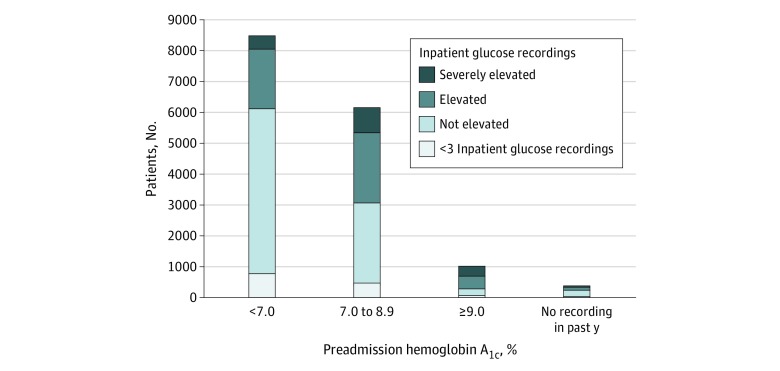
Association Between Inpatient Blood Glucose Recordings and Preadmission Outpatient Hemoglobin A_1c_ Level Preadmission hemoglobin A_1c_ was measured using most recent hemoglobin A_1c_ laboratory value collected within 1 year preceding hospitalization. Inpatient blood glucose control was defined by the number of elevated blood glucose recordings as severely elevated (≥3 recordings of ≥300 mg/dL [to convert to millimoles per liter, multiply by 0.0555]), moderately elevated (≥3 recordings of ≥200 mg/dL without meeting criteria for severely elevated), or not elevated. Preadmission hemoglobin A_1c_ was found to be associated with inpatient blood glucose categories (*P* < .001 using a χ^2^ test). To convert hemoglobin A_1c_ to proportion of total hemoglobin, multiply by 0.01.

Prior to hospitalization, 8535 patients (53%) had an HbA_1c_ level less than 7.0%, 6199 (38%) had an HbA_1c_ level between 7.0% and 8.9%, 1044 (6%) had an HbA_1c_ level greater than 9.0%, and 400 (2%) did not have HbA_1c_ level measured. During hospitalization, 1626 patients (10%) had severely elevated inpatient blood glucose levels (≥3 recordings >299 mg/dL) and 4724 (29%) had moderately elevated inpatient blood glucose levels (≥3 recordings >199 mg/dL). Elevated inpatient blood glucose levels were more common in patients with elevated preadmission HbA_1c_ (*P* < .001 for trend); however, 37% of patients with elevated inpatient blood glucose levels had a preadmission HbA_1c_ level less than 7.0% (Figure 1).

### Diabetes Medication Intensifications

A total of 1626 patients (10%) were discharged with intensified diabetes medications and 1301 (8%) were discharged with intensified high-risk diabetes medications (Table 2). The most common intensifications were insulin additions, which occurred in 781 hospitalizations (5%). Intensifications of sulfonylureas occurred in 557 hospitalizations (3%); however, 94 patients with new sulfonylureas were noted to have filled a different sulfonylurea prior to admission, so these changes may reflect therapeutic substitutions. Intensifications of metformin were also common, occurring in 382 hospitalizations (2%), while intensifications of all other medication classes were infrequent.

**Table 2.** Diabetes Medication Intensifications

Intensification	Patients, No. (%) (N = 16 178)
Any intensification	1626 (10)
Intensification of high-risk medications^a^	1301 (8)
Insulin	
Any	781 (5)
Long-acting insulin start	678 (4)
Short-acting insulin start	307 (2)
Both long and short acting insulin start	204 (1)
Sulfonylureas	
Any	557 (3)
New start^b^	425 (3)
Dose increase	132 (1)
Metformin	
Any	382 (2)
New start	298 (2)
Dose increase	84 (1)
Other	
Any^c^	38 (0)
Dipeptidyl peptidase-4 inhibitors	17 (0)
α-Glucosidase inhibitors	11 (0)
Thiazolidinediones	8 (0)
Meglitinides	4 (0)
Glucagon-like peptide-1 agonists	0

^a^ High-risk medications were defined as classes with increased risk of hypoglycemia and included insulins and sulfonylureas.

^b^ Ninety-four patients with new sulfonylureas were noted to have filled a different sulfonylurea prior to admission.

^c^ All other diabetes medication intensifications were new prescriptions with the exception of 3 patients receiving dose increases of α-glucosidase inhibitors.

**Figure 2.**
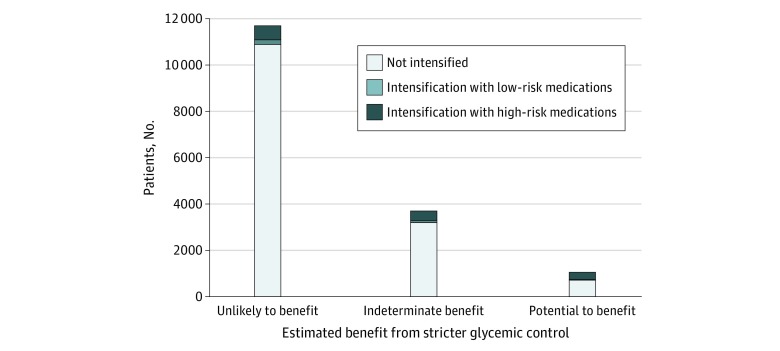
Intensification of Diabetes Medications by Estimated Likelihood to Benefit High-risk intensifications included addition of insulin and/or addition or dose increase of sulfonylurea medications. Likelihood of benefit from diabetes medication intensification was estimated using preadmission hemoglobin A_1c_ and estimated life expectancy. Patients were categorized as having potential to benefit if their preadmission hemoglobin A_1c_ level was greater than 9.0% (to convert to proportion of total hemoglobin, multiply by 0.01). Patients were categorized as unlikely to benefit if their preadmission hemoglobin A_1c_ level was less than 7.5% regardless of life expectancy or if their preadmission hemoglobin A_1c_ level was less than 9.0% and their estimated life expectancy was less than 5 years. All others were classified as having indeterminate benefit (eTable 1 in the Supplement). Estimated likelihood of benefit was found to be associated with intensification category (*P* < .001 using a χ^2^ test).

The majority of patients (77% [12 441 of 16 178]) were classified as being unlikely to benefit from more intensive diabetes control based on preadmission diabetes control and life expectancy, 6% (1044 of 16 178) were categorized as being likely to benefit, and 17% (2693 of 16 178) were categorized as having indeterminate benefit (eTable 1 in the Supplement). Figure 2 shows that 49% of patients receiving intensifications (791 of 1626) were classified as unlikely to benefit, 20% (329 of 1626) as likely to benefit, and 31% (506 of 1626) as having indeterminant benefit. Among patients receiving intensifications with high-risk medications, 45% (588 of 1301) were classified as being unlikely to benefit, 23% (305 of 1301) as likely to benefit, and 31% (408 of 1301) as having indeterminant benefit.

### Probability of Intensification by Preadmission and Inpatient Diabetes Control

**Figure 3.**
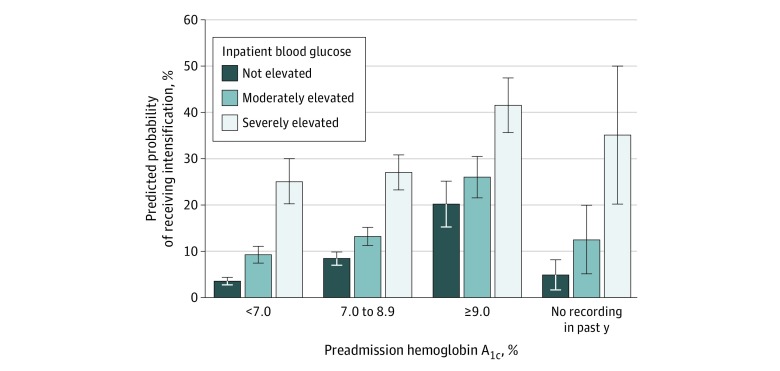
Predicted Probability of Receiving a Diabetes Medication Intensification at Discharge, by Inpatient and Outpatient Glucose Control Preadmission hemoglobin A_1c_ was measured using the most recent laboratory value collected within 1 year preceding hospitalization. Inpatient blood glucose control was defined by the number of elevated blood glucose recordings as severely elevated (≥3 recordings of ≥300 mg/dL [to convert to millimoles per liter, multiply by 0.0555]), moderately elevated (≥3 recordings of ≥200 mg/dL without meeting criteria for severely elevated), or not elevated. Predicted probabilities were estimated following mixed-effect logistic regression accounting for age, sex, race/ethnicity, income, Charlson Comorbidity Index score, length of stay, primary discharge diagnosis, year, hospital training status, receipt of steroids during hospitalization, preadmission hemoglobin A_1c_, inpatient blood glucose level, an interaction term for preadmission hemoglobin A_1c_ and inpatient blood glucose level, and random effects to account for clustering by Veterans Health Administration hospital. Error bars indicate 95% CIs. To convert hemoglobin A_1c_ to proportion of total hemoglobin, multiply by 0.01.

Figure 3 shows the predicted probabilities of receiving intensifications by both preadmission and inpatient diabetes control and demonstrates that each preadmission HbA_1c_ category was associated with increasingly greater predicted probabilities of receiving intensifications based on patients’ inpatient blood glucose recordings. For example, among patients with a preadmission HbA_1c_ level less than 7.0%, the predicted probability of being discharged with intensifications was 4% (95% CI, 3%-4%) for those without elevated inpatient blood glucose levels and 21% (95% CI, 15%-26%) for those with severely elevated inpatient blood glucose levels.

Notably, both elevated preadmission HbA_1c_ level and inpatient glucose control were associated with receipt of intensifications, with the odds of receiving intensifications increasing with increasing severity of both inpatient blood glucose level and outpatient and preadmission HbA_1c_ level. However, 30% of patients receiving intensifications (486 of 1626) had tightly controlled HbA_1c_ prior to hospitalization (eTable 2 in the Supplement).

### Other Factors Associated With Diabetes Medication Intensifications

eTable 3 in the Supplement shows the adjusted odds ratios (ORs) for each covariate included in the model. Receiving no diabetes medications prior to hospitalization was associated with higher odds of receiving intensifications compared with taking 1 medication on admission (OR, 1.84; 95% CI, 1.58-2.15). Other hospitalization factors associated with increased odds of receiving intensifications were receipt of inpatient corticosteroids (OR, 1.24; 95% CI, 1.04-1.49) and longer length of stay (OR, 1.41; 95% CI, 1.12-1.78 for length of stay ≥14 days compared with <3 days). Hospitalization with heart failure (OR, 1.46; 95% CI, 1.15-1.84) and coronary artery disease (OR, 1.35; 95% CI, 1.06-1.73) was associated with increased odds of receiving intensifications compared with a reference condition of pneumonia. Preadmission medication adherence was not associated with receiving diabetes medication intensifications.

## Discussion

In this national retrospective cohort study of older adults with diabetes who were hospitalized for common medical conditions, we found that intensified diabetes medications were prescribed at discharge for 1 in 10 patients, most of whom we estimated were unlikely to benefit from stricter glycemic control. Most intensifications were for insulins or sulfonylureas, classes with the highest risk of hypoglycemia,^23,24^ and intensifications commonly occurred in patients previously not taking diabetes medications. Elevated inpatient blood glucose recordings were associated with discharge with intensifications even in patients with previously well-controlled outpatient HbA_1c_ values. These findings indicate that half of older adults receiving diabetes medication intensifications at hospital discharge may be at high risk of overtreatment while 20% may have potential to benefit.

In the hospital setting, many factors may cause blood glucose levels to fluctuate. Patients may experience physiologic stress hyperglycemia related to acute illness,^25^ be given medications that raise blood glucose levels (eg, corticosteroids or intravenous solutions containing dextrose), and have different dietary patterns than they would at home. Both acute kidney injury and inability to take pills by mouth are common in hospitalized older adults and may lead inpatient clinicians to withhold outpatient diabetes medications. For this reason, temporary inpatient sliding scale insulin protocols have become common despite risks of hypoglycemia.^26^ Our finding that nearly half of intensifications were for new insulin, primarily short-acting insulins, may represent high-risk carryover of inpatient regimens to the outpatient setting. While we found that most patients were unlikely to benefit from intensifications, for patients with both severe inpatient hyperglycemia and uncontrolled outpatient blood glucose levels who may benefit from intensifications, simpler guideline-based insulin regimens, starting with single dose long-acting agents, is likely to lessen hypoglycemia risks compared with more complex short-acting or multidrug insulin regimens.^27^

We found that one-third of patients with elevated inpatient blood glucose recordings had tightly controlled preadmission HbA_1c_, indicating that inpatient hyperglycemia is not a reliable marker to guide adjustments to outpatient diabetes regimens. Despite this, our findings indicate that inpatient blood glucose elevations frequently lead clinicians to intensify outpatient diabetes treatment, which may lead to unnecessarily intensive treatment. Prior evidence suggests that as many as 1 in 5 older adults receive potentially unnecessary intensive diabetes treatment, which is associated with increased risks for severe hypoglycemia.^8^ Evidence from the VHA and Medicare indicates that intensive treatment is common in older adults with medically complex conditions.^8,28,29,30^ While guidelines for long-term HbA_1c_ targets for older adults are conflicting, there is consensus on the need to balance the benefits of intensive HbA_1c_ control with the risks of hypoglycemia, particularly in patients with limited life expectancy.^10^ Our findings that nearly half of patients receiving intensifications were unlikely to benefit owing to limited life expectancy or already achieving goal HbA_1c_ and that only 20% of patients with potential to benefit from intensifications received them indicate that likelihood for long-term benefit did not strongly influence intensification decisions made during hospitalization.

Even in patients who may benefit from stricter blood glucose control, it is unclear whether hospitalization is the right time to intensify outpatient therapy. Older adults are at an elevated risk for readmission and adverse drug events in the period after hospitalization^31,32,33^ and medication confusion at discharge is common and may be related to poor communication, medication reconciliation errors, delirium, or multiple changes. Few studies have examined posthospitalization changes in medications for chronic disease. One study^34^ of patients filling insulin following discharge observed a subsequent increased risk of death and readmissions; however, this study lacked information on patients’ inpatient or outpatient glycemic control. Another study^4^ examining the antihypertensive intensifications at discharge found increased risks of adverse events and readmissions. Thus, additional information is needed on the long-term outcomes of intensifying diabetes medications at discharge. In the meantime, if there is concern about persistent hyperglycemia after the patient leaves the hospital, for most patients, a safer course than intensifying medications may be to focus on communicating with outpatient providers to inform decision-making following recovery from hospitalization. For patients with severe hyperglycemia on the day of discharge, ensuring close outpatient follow-up, prioritizing restarting home medications, and, if medication intensification is warranted, choosing agents with less hypoglycemia risk may minimize potential overtreatment. For both patient groups, using hospital resources to focus on diabetes education has demonstrated long-term benefits^35,36^ and may be preferable to medication intensification.

### Limitations

Our study has several limitations. Pharmacy records allow for identification of dose changes for oral medications but do not accurately reflect changes to insulin dosing; thus, we excluded patients taking insulin prior to hospitalization. We were unable to assess the frequency of medication discontinuations, leaving the possibility that some patients receiving intensifications may have had other diabetes medications reduced. Our study was conducted in the national VHA health system, which serves a higher proportion of men and patients with multiple chronic conditions and uses a national pharmacy benefit management system, which may limit variation in medication selection. However, as this study examined prescribing practices and the VHA is a primary training site for US graduate medical education, practices developed by trainees in the VHA may be carried on to other care settings. Our study examined only older adults; thus, our findings are not generalizable to younger populations. Since our study period, new classes of diabetes medications have been introduced, which may reduce the generalizability of our findings to current practice patterns.

## Conclusions

This study found that 1 in 10 hospitalized older adults with diabetes was discharged with intensifications to their outpatient diabetes medication regimens, and most intensifications were initiations of new insulins and sulfonylureas. Nearly half of older adults receiving intensifications had limited life expectancy or had already achieved an outpatient HbA_1c_ level less than 7.5%; thus, they were unlikely to benefit from this additional pharmacotherapy that appears to be prescribed largely in response to elevated inpatient blood glucose levels. Conversely, among patients with potential to benefit from stricter glycemic control, only 20% received intensifications, although even in this population, the clinical outcomes of intensifications made during recovery from acute illness are unknown. Improving diabetes care for hospitalized older adults will require efforts to move beyond treating elevated inpatient numbers and toward patient-centered decision-making that considers long-term benefits and the risks of potentially unnecessary medication intensifications.

## Notes

Supplement. **eFigure.** Cohort Construction Flowchart **eTable 1.** Likelihood of Cohort Patients to Benefit From Stricter Glycemic Control **eTable 2.** Rates of Diabetes Medication Intensification in Older Adults Following Hospitalization, by Inpatient and Outpatient Blood Glucose Control **eTable 3. **Predictors of Diabetes Medication Intensification in Older Adults Following Hospitalization
