# Error in Abstract

**DOI:** 10.1001/jamanetworkopen.2020.6250

**Published:** 2020-04-20

In the Original Investigation titled “Prevalence of Diabetes Medication Intensifications in Older Adults Discharged From US Veterans Health Administration Hospitals,”^1^ published March 24, 2020, there was an error in the dates of the study given in the Design, Setting, and Participants section of the Abstract. These dates appeared as 2012 to 2013 but should have appeared as 2011 to 2013. This article has been corrected.^1^
