# Supplementary material for: Prevalence of Diabetes Medication Intensifications in Older Adults Discharged From US Veterans Health Administration Hospitals
Source: JAMA Netw Open. 2020 Mar 24;3(3):e201511. doi: 10.1001/jamanetworkopen.2020.1511 (PMC7093767; doi:10.1001/jamanetworkopen.2020.1511)
Supplement: Supplement. — eFigure. Cohort Construction Flowchart eTable 1. Likelihood of Cohort Patients to Benefit From Stricter Glycemic Control eTable 2. Rates of Diabetes Medication Intensification in Older Adults Following Hospitalization, by Inpatient and Outpatient Blood Glucose Control eTable 3. Predictors of Diabetes Medication Intensification in Older Adults Following Hospitalization [file jamanetwopen-3-e201511-s001.pdf]

Supplementary Online Content

Anderson TS, Lee S, Jing B, et al. Prevalence of diabetes medication intensifications in older adults discharged from US Veterans Health Administration hospitals. *JAMA Netw Open*. 2020;3(3):e201511. doi:10.1001/jamanetworkopen.2020.1511

eFigure. Cohort Construction Flowchart

eTable 1. Likelihood of Cohort Patients to Benefit From Stricter Glycemic Control

eTable 2. Rates of Diabetes Medication Intensification in Older Adults Following Hospitalization, by Inpatient and Outpatient Blood Glucose Control

eTable 3. Predictors of Diabetes Medication Intensification in Older Adults Following Hospitalization

This supplementary material has been provided by the authors to give readers additional information about their work.

© 2020 Anderson TS et al. *JAMA Network Open.*

eFigure: Cohort construction flowchart

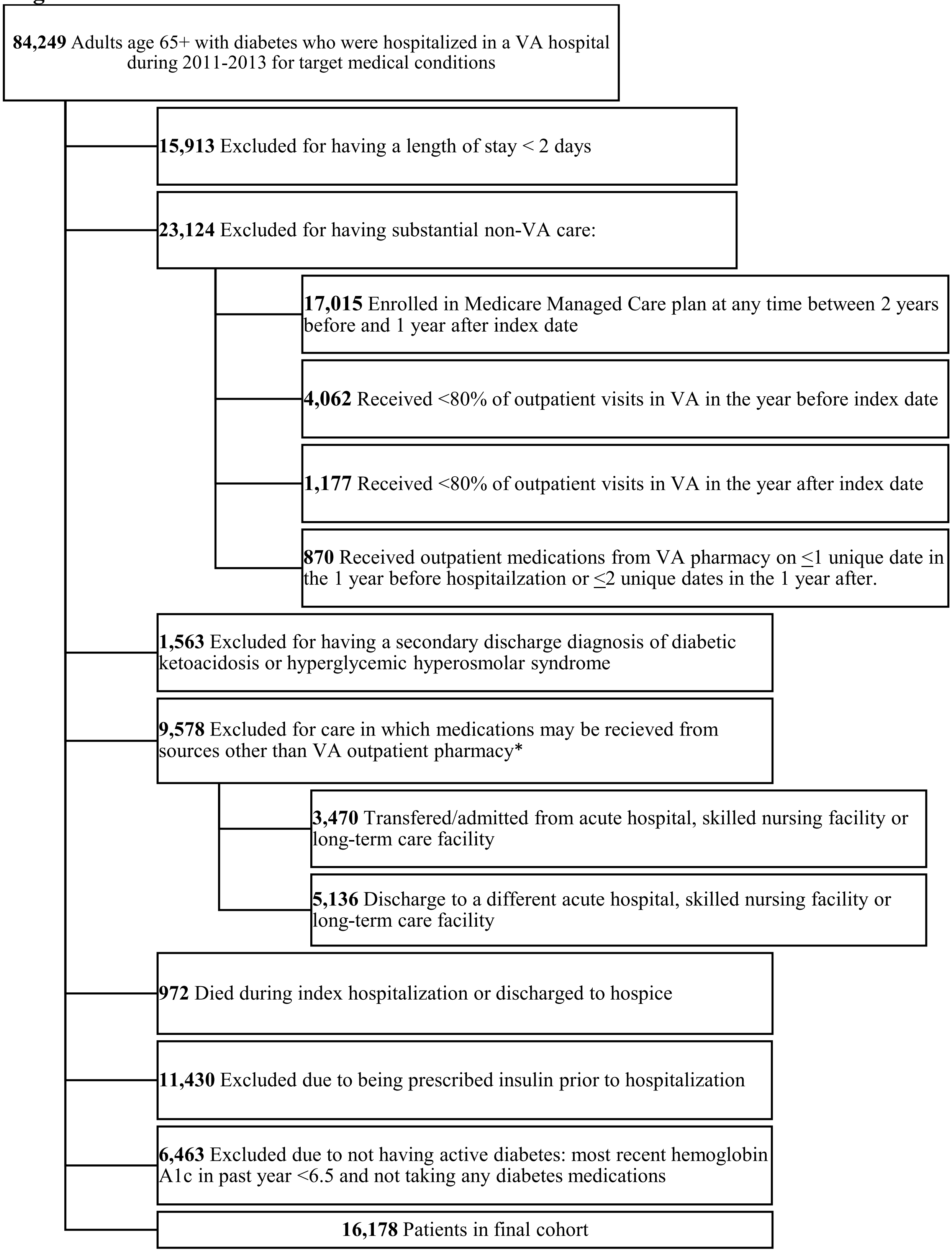

© 2020 Anderson TS et al. *JAMA Network Open.*

eTable 1. Likelihood of cohort patients to benefit from stricter glycemic control

	**Preadmission hemoglobin A1c control**			
**Life expectancy**	<7.5%	7.5% to 8.9%	>9.0%	Not measured in prior year
>10 years	4,984	1,415	511	155
5 to 10 years	3,337	878	309	128
<5 years	3,169	951	224	117

Note: Likelihood of benefit from diabetes medication intensification defined by preadmission hemoglobin A1c and estimated life expectancy and color coded as follows:
Orange boxes – Unlikely to benefit from stricter glycemic control
Grey boxes – indeterminate likelihood of benefit
Blue boxes – likely to benefit stricter glycemic control

© 2020 Anderson TS et al. *JAMA Network Open.*

eTable 2. Rates of diabetes medication intensification in older adults following hospitalization, by inpatient and outpatient blood glucose control

		Odds Ratio (95% CI)	
	Intensification, No. (%)	Unadjusted	Adjusted
Overall cohort (n=16,178)	1,626 (10.5)		
Preadmission hemoglobin A1c [a]			
Tightly-controlled: <7.0% (n=8,535)	486 (5.7)	1 [Reference]	1 [Reference]
Controlled: 7.0 to 8.9% (n=6,199)	777 (12.5)	2.39 (2.12 to 2.70)	2.46 (2.03 to 2.98)
Elevated: ≥9.0% (n=1,044)	329 (31.5)	7.79 (6.63 to 9.16)	7.06 (5.01 to 9.94)
Not measured (n=400)	34 (8.5)	1.55 (1.07 to 2.23)	1.37 (0.69 to 2.72)
Inpatient blood glucose [b]			
Not elevated (n=8,407)	549 (5.8)	1 [Reference]	1 [Reference]
Moderately elevated (n=4,724)	596 (11.9)	2.48 (2.18 to 2.83)	2.74 (2.20 to 3.42)
Severe elevated (n=1,620)	481 (28.4)	7.78 (6.69 to 9.06)	9.46 (7.11 to 12.59)

Note: Adjusted odds ratios estimated using mixed effect logistic regression accounting for age, gender, race, income, Charlson comorbidities, length of stay, primary discharge diagnosis, year, hospital training status, receipt of steroids during hospitalization, preadmission HbA1c, inpatient blood glucose, an interaction term for preadmission HbA1c and inpatient blood glucose, and random effects to account for clustering by VA hospital.

[a] Preadmission hemoglobin A1c measured using most recent hemoglobin A1c laboratory value collected within 1 year preceding hospitalization and defined as: *tightly-controlled* if HbA1c <7.0%, *controlled* if HbA1c 7.0% to 8.9%, or *elevated* if HbA1c >=9.0%, or not measured if no HbA1c laboratory was available in the year preceding hospitalization.

[b] Inpatient blood glucose control defined by number of elevated blood glucose recordings and defined as: *severely elevated* if >=3 blood glucose recordings of >=300 mg/dL; *moderately elevated* if >=3 blood glucose recordings of >=200 mg/dL without meeting criteria for *severely elevated*, or *not elevated.* For patients with missing or fewer than 3 blood glucose recordings, multiple imputation was conducted.

© 2020 Anderson TS et al. *JAMA Network Open.*

eTable 3. Predictors of diabetes medication intensification in older adults following hospitalization

Characteristic	Not intensified (N=14552)	Intensified (N=1626)	Adjusted OR (95% CI)
**Demographics and comorbidities**			
Age			
65-74	7,932	987	1 [Reference]
75-84	4,608	475	0.88 (0.77 to 0.98)
≥85	2,012	164	0.65 (0.53 to 0.78)
Female	251	32	0.98 (0.65 to 1.47)
Race/ethnicity			
White	11,859	1,279	1 [Reference]
Black	2,061	275	1.12 (0.95 to 1.32)
Hispanic	210	15	0.81 (0.46 to 1.43)
Other	422	57	1.12 (0.82 to 1.52)
Income	26668 (467998)	26517 (44822)	1.00 (1.00 to 1.00)
**Hospitalization characteristics**			
Year			
2011	5,705	665	1 [Reference]
2012	4,613	488	0.90 (0.78 to 1.03)
2013	4,234	473	0.91 (0.79 to 1.04)
Teaching hospital	13,089	1,462	0.78 (0.57 to 1.06)
Length of stay, days			
2	3,945	323	1 [Reference]
3-4	5,041	489	1.08 (0.93 to 1.24)
5-7	3,265	409	1.20 (1.01 to 1.43)
8-13	1,645	268	1.31 (1.10 to 1.56
≥14	656	137	1.41 (1.12 to 1.78)
**Preadmission laboratories**			
Hemoglobin A1c			
Tightly-controlled: <7.0%	8,049	486	1 [Reference]
Controlled: 7.0 to 8.9%	5,422	777	2.46 (2.03 to 2.98)
Elevated: ≥9.0%	715	329	7.06 (5.01 to 9.94)
Not measured	366	34	1.37 (0.69 to 2.72)
eGFR, ml/min/1.73 m^2			
>90	1,977	180	1 [Reference]
60-89	5,671	583	1.06 (0.86 to 1.30)
45-59	2,884	353	1.24 (0.96 to 1.60)
30-44	1,584	178	1.06 (0.74 to 1.52)
15-30	490	63	0.96 (0.69 to 1.34)
<15	209	28	1.21 (0.61 to 2.40)
**Inpatient laboratories**			
Inpatient blood glucose			
Not elevated	7,920	487	1 [Reference]
Moderately elevated	4,163	561	2.74 (2.20 to 3.42)
Severe elevated	1,163	457	9.46 (7.11 to 12.59)
Discharge eGFR, ml/min/1.73 m^2			
>90	2,541	238	1 [Reference]
60-89	5,509	564	1.07 (0.89 to 1.29)
45-59	2,671	347	1.25 (0.98 to 1.59)
30-44	1,417	191	1.29 (0.91 to 1.84)
15-30	508	78	1.33 (0.99 to 1.80)

© 2020 Anderson TS et al. *JAMA Network Open.*

<15	232	34	1.28 (0.69 to 2.36)
**Medication use**			
Admission medication count	9.2 (4.9)	7.9 (4.7)	0.99 (0.98 to 1.00)
No. admission diabetes medications			
0	3,505	648	1.84 (1.58 to 2.15)
1	7,635	628	1 [Reference]
2	3,056	294	0.76 (0.62 to 0.93)
3	338	52	0.78 (0.49 to 1.26)
≥4	18	4	2.76 (0.67 to 11.37)
Prior diabetes medication adherence			
Proportion of days covered <80%	3,354	346	1.07 (0.89 to 1.29)
Proportion of days covered ≥80%	7,693	632	Reference
Any receipt of inpatient corticosteroids	1,551	259	1.24 (1.04 to 1.49)
**Comorbidities**			
Congestive heart failure	6,054	718	0.94 (0.82 to 1.08)
Renal disease	4,513	548	0.85 (0.73 to 0.99)
Cerebrovascular accident	3,784	433	1.05 (0.91 to 1.21)
Prior myocardial infarction	2,871	333	0.93 (0.79 to 1.09)
Malignancy	3,249	325	0.99 (0.86 to 1.14)
Dementia	472	47	0.86 (0.61 to 1.20)
**Admission diagnoses**			
Pneumonia	1,721	172	1 [Reference]
Arrhythmia	206	11	0.69 (0.36 to 1.34)
Asthma	72	12	1.28 (0.64 to 2.52)
Chronic obstructive pulmonary disease	1,344	187	1.12 (0.87 to 1.43)
Chest pain	618	46	1.00 (0.69 to 1.45)
Conduction disorders	1,491	126	1.11 (0.86 to 1.45)
Coronary artery disease	1,602	193	1.35 (1.06 to 1.73)
Acute myocardial infarction	861	110	1.35 (0.99 to 1.84)
Heart failure	2,258	295	1.46 (1.15 to 1.84)
Heart valve disorders	281	31	1.35 (0.87 to 2.08)
Sepsis	259	26	1.06 (0.66 to 1.70)
Skin infection	1,245	132	1.01 (0.77 to 1.31)
Stroke	701	90	1.16 (0.84 to 1.60)
Transient ischemic attach	243	20	1.04 (0.61 to 1.76)
Urinary tract infection	1,298	131	1.08 (0.83 to 1.41)
Venous thromboembolism	352	44	1.25 (0.85 to 1.83)

© 2020 Anderson TS et al. *JAMA Network Open.*
